# Chemical synthesis of Nd_*x*_Co_1−*x*_Fe_2_O_4_ hybrid nanoparticles for permanent magnet applications: structural, magnetic and electrical properties

**DOI:** 10.1039/d5na00197h

**Published:** 2025-03-18

**Authors:** Saleh M. Matar, Galal H. Ramzy, Muhammad Arif, Ibrahim M. Maafa, Ayman Yousef, Nasser Zouli, Ahmed F. F. Abouatiaa, Abdel Samed M. Adam, Isam Y. Qudsieh, Ahmed I. Ali, Elbadawy A. Kamoun, Amr Ali

**Affiliations:** a Department of Chemical Engineering, College of Engineering and Computer Sciences, Jazan University Jazan 45142 Saudi Arabia; b Physics Department, Faculty of Science, Cairo University Giza 12613 Egypt; c Department of Applied Physics, Integrated Education Institute for Frontier Science and Technology and Institute of Natural Sciences, Kyung Hee University Yongin 17104 Republic of Korea; d Basic Science Department, Faculty of Technology and Education, Helwan University Saray–El Qoupa, El Sawah Street 11281 Cairo Egypt Ahmed_Ali_2010@techedu.helwan.edu.eg; e Department of Mechanical Engineering (Integrated Engineering Program), Kyung Hee University 1732 Deogyeong-Daero Yongin Gyeonggi 17104 Republic of Korea; f Department of Chemistry, College of Science, King Faisal University Al-Ahsa 31982 Saudi Arabia ekamoun@kfu.edu.sa badawykamoun@yahoo.com; g Polymeric Materials Research Dep., Advanced Technology and New Materials Research Institute (ATNMRI), City of Scientific Research and Technological Applications (SRTA-City) New Borg Al-Arab City 21934 Alexandria Egypt; h Production Technology Department, Faculty of Technology & Education, Sohag University 82524 Sohag Egypt

## Abstract

Nd-doped CoFe_2_O_4_ spinel ferrites were synthesized *via* the sol–gel method, confirming a cubic spinel structure. Increasing Nd concentration expanded the lattice parameter (8.3900–8.4231 Å) and unit cell volume while reducing grain size. FT-IR analysis validated the spinel phase. Nd doping enhanced the dielectric constant by affecting space charge polarization and charge hopping, with conductivity following a Debye-type relaxation mechanism. Cole–Cole plots indicated grain boundary effects and polaron hopping conduction. Magnetic properties improved with Nd^3+^ content, with *M*_s_ and *H*_c_ reaching 5.621 emu g^−1^ and 143.43 Oe at 4% doping. A transition from an antiferromagnetic to a ferromagnetic state was observed, with a high Curie temperature (*T*_m_) of 292 °C, confirming a stable ferromagnetic phase. These findings highlight Nd-doped CoFe_2_O_4_ as a promising candidate for permanent magnet applications.

## Abstract 2

Nd-doped CoFe_2_O_4_ spinel ferrites were synthesized *via* the sol–gel method, confirming a cubic spinel structure.

## Graphical abstract

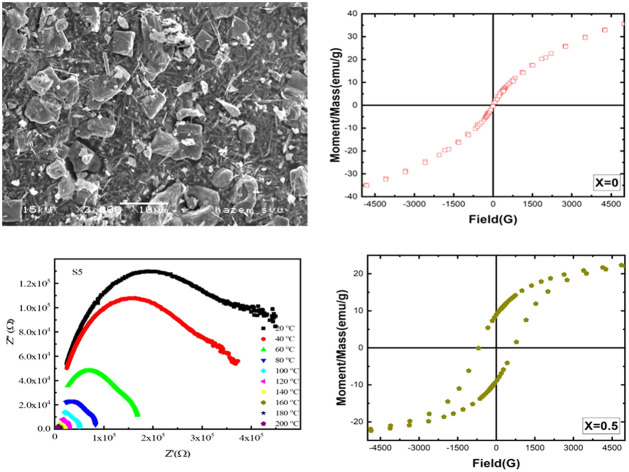

## 1. Introduction

Recently, ferrites with a spinel structure have attracted significant attention due to their wide range of potential applications, including magnetic fluids, high-density magnetic recording, data storage devices, spintronics, solar cells, sensors, and catalysis.^1,2^ In the spinel cubic structure, oxygen ions are dispersed among 64 tetrahedral and 32 octahedral sites. The metal cations can occupy 8 A-sites and 16 B-sites, with migrating cations potentially filling up the vacant positions. Several factors including growth conditions such as the types of elements in the composite, doping, temperature, pressure, sintering time, fabrication method, and cation distribution between A and B-sites can all have an impact on the physical properties of the spinel structure.^3^ The most well-known member of the iron oxide family is cubic spinel structured cobalt ferrite (CoFe_3_O_4_) and is a promising candidate for a variety of magnetic recording applications because of its exceptional chemical and physical durability.^4–6^ Because of its exceptional electrical and magnetic properties, strong magneto-crystalline anisotropy, and chemical, mechanical, and thermal stability, CoFe_2_O_4_ is one of the most significant compounds that are being thoroughly researched.^4,7^ In the CoFe_2_O_4_ spinel ferrite structure, both Fe^3+^ and Co^2+^ ions occupy tetrahedral and octahedral interstitial sites.^8–10^ Additionally, the structure features a face-centered cubic (FCC) with a close-packed configuration of oxygen ions. CoFe_2_O_4_ offers several advantages because of the ferromagnetic state that arises from the super-exchange interactions between Fe^3+^ and Co^2+^ ions *via* O^2−^ ions.^11^ However, an exceptionally high crossover rate is still a significant obstacle, which might be overcome by doping metal ions. In particular, rare earth ions, known for their high resistivity, are useful dopants for spinel ferrites.^12–14^ According to previously published reports, neodymium (Nd) doping into TiO_2_ increases carrier longevity, enabling charge transfer and serving as direct trapping spots.^15^ Owing to their wide range of applications, several methods including co-precipitation, combustion, sol–gel, solid–state reaction, and precipitation have been employed.^16–18^ Among these, the sol–gel technique offers a more simple and effective way to produce CoFe_2_O_4_ nanoparticles.^19^ Ni-doped ferrite magnetic nanoparticles (Ni_*x*_Co_1−*x*_Fe_2_O_4_) have been synthesized, and their magnetic properties have been investigated at room temperature. Magnetic hysteresis loop measurements revealed that both the saturation magnetization (*M*_s_) and coercivity (*H*_c_) decreased linearly with increasing Nd^3+^ content.^7^ Likewise, magnetic nanoparticle Co_0.5_Nd_0.5_Fe_2_O_4_ spinel ferrites have been synthesized by means of the co-precipitation technique and their room temperature magnetic characteristics were investigated, and it was observed that with greater Nd^+^ doping levels, *M*_s_ reduced but *H*_c_ increased.^20,21^ In addition, Yadav *et al.*^3^ synthesized nano-crystalline ferrite particles (CoFe_2−*x*_Nd_*x*_O_4_ at lower doping levels) and observed a simultaneous increase in both *M*_s_ and *H*_c_ with the addition of Nd^3+^ ions. Furthermore, the doping effect of elements like La, Er, and Ce on the ferrites has been investigated.^22,23^ It has been noted that adding these ions in place of Fe^3+^ leads to strain and structural alterations, which in turn weakens the particle's physical characteristics. Consequently, it is extremely important to monitor how Nd^3+^ doping in the B-site affects the structural, electrical, and magnetic characteristics of CoFe_2_O_4_ ferrite particles.^24^

Nd^3+^ doped CoFe_2_O_4_ has significantly influenced its structural, magnetic, and electrical properties, due to its large ionic radius and trivalent nature. The incorporation of Nd^3+^ into the spinel lattice primarily affects cation distribution, resulting in lattice distortion and potential phase transformation. Nd^3+^ preferentially occupies octahedral sites, causing partial inversion of the spinel structure and altering Co^2+^–Fe^3+^ super-exchange interactions. This redistribution of cations modulates the material's magnetic behavior, potentially reducing saturation magnetization due to the non-magnetic nature of Nd^3+^ and modifying coercivity based on strain-induced anisotropy changes.^25,26^

The electrical properties of CoFe_2_O_4_ are also impacted by Nd doping, in which charge compensation mechanisms introduce oxygen vacancies, influencing conductivity and dielectric properties. The presence of these vacancies enhances leakage current characteristics, improving energy storage performance. Furthermore, dielectric constant variations suggest improved charge carrier mobility, which can be beneficial for high-frequency applications.^27^

Structurally, Nd doping induces strain within the CoFe_2_O_4_ lattice, potentially leading to phase segregation at higher doping concentrations. The formation of secondary phases, such as NdFeO_3_ or NdCoO_3_, is observed when the solubility limit is exceeded, affecting the thermal and mechanical stability of the material. This phase evolution highlights the complex interplay between ionic size effects, charge balance, and structural integrity in Nd-doped CoFe_2_O_4_.^28,29^

Neodymium (Nd), a lanthanide element, is widely used in the production of permanent magnets,^30^ which have diverse applications across various industries.^31^ Nd-based magnets are essential for hard disk drives, mobile phones, televisions, magnetic separators, and medical devices like magnetic resonance imaging (MRI) machines.^32–34^ However, developing cost-effective and environmentally sustainable methods for recovering neodymium from aquatic environments remains a critical challenge. The sol–gel method is ideal for synthesizing Nd-doped CoFe_2_O_4_, due to its high chemical homogeneity, precise stoichiometry, and controlled structural properties. It enables uniform ion mixing, minimizing phase segregation and ensuring material quality. Using metal nitrates or acetates as precursors allows accurate dopant control, preserving the spinel structure. Its low processing temperature reduces energy consumption and limits grain growth, resulting in fine nanoparticles with enhanced magnetic and electrical properties. The method also offers excellent phase purity, crystallinity, and tunable particle size, making it suitable for high-frequency applications and scalable for large-scale production.^35^ Based on this, we believe that Nd-doping in A-sites could enhance the performance of CoFe_2_O_4_, making it promising for advanced applications. This doping strategy significantly modifies structural, electrical, and magnetic properties, benefiting nanoelectronics. This study explores the impact of Nd doping on the structural, magnetic, and electrical properties of CoFe_2_O_4_, revealing key transformations that enhance functionality. Structurally, Nd incorporation expands the lattice, induces strain, and influences phase stability. It suppresses grain growth and promotes secondary phase formation at high doping levels. Magnetically, Nd alters super-exchange interactions, leading to spin canting, reduced saturation magnetization, and variable coercivity. Increased magnetic anisotropy highlights its tunability for applications. Electrically, Nd suppresses Fe^2+^/Fe^3+^ hopping, reducing conductivity while improving leakage current and dielectric properties, optimizing energy storage density and efficiency.

In this study, we investigate Nd-doping in the A-site of cobalt ferrite (Nd_*x*_Co_1−*x*_Fe_2_O_4_) nanoparticles synthesized *via* the sol–gel combustion method. Various characterization techniques were employed to analyze their magnetic and dielectric properties. The novelty of this work lies in Nd-doping, which has been less explored in cobalt ferrite. Nd^3+^ incorporation in the A-site enhances dielectric properties by reducing losses, improving high-frequency performance and optimizing magnetic behavior by influencing cation distribution within the spinel structure. This synergistic effect enhances electrical conductivity and overall material performance, making Nd-doped cobalt ferrite promising for applications in sensors, catalysis, high-frequency devices, and nanoelectronics. This research provides a foundation for the practical use of such nanoparticles.

## 2. Materials and methods

### 2.1. Materials

High purity cobalt nitrate (Co(NO_3_)_2_·6H_2_O (3N)) and iron nitrate (Fe(NO_3_)_3_·9H_2_O (3N)) were purchased from Al-Gomhouria Chemical Company in Cairo, Egypt. Neodymium nitrate (Nd(NO_3_)_3_·6H_2_O (3N)) was purchased from Sigma-Aldrich, Germany.

### 2.2. Preparation of samples

**Fig. 1:**
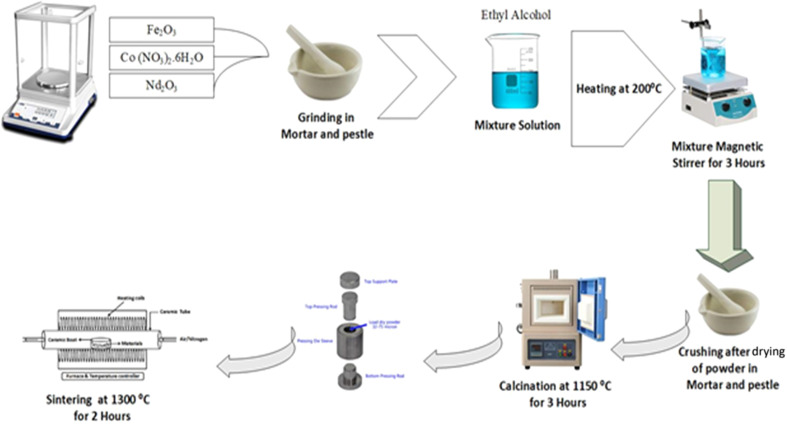
Schematic representation of Nd^3+^ doped-CoFe_2_O_4_ solid solution prepared by the sol–gel technique.

Nd_*x*_Co_1−*x*_Fe_2_O_4_ (*x* = 0–0.5) powders, as listed in Table 1, were prepared using the sol–gel method. The detailed procedure, shown in Fig. 1, is as follows: first, stoichiometric amounts of high-purity cobalt nitrate (Co(NO_3_)_2_·6H_2_O, 3N), iron nitrate (Fe(NO_3_)_3_·9H_2_O, 3N), and neodymium nitrate (Nd(NO_3_)_3_·6H_2_O, 3N) were measured using a digital balance and mixed thoroughly with a mortar and pestle for 30 minutes to prepare the precursor. In the next step, the raw materials were mixed with ethyl alcohol as a solvent and sonicated for 20 minutes. The solution was then heated on a hot plate at 85 °C for 2 hours under magnetic stirring. After preparing the precursor solution, precipitation was induced by adjusting pH to 7 with an ammonium hydroxide solution (NaOH, 6 mol L^−1^) under constant stirring. The precipitates were allowed to settle and were subsequently collected by vacuum filtration. To ensure purity, the precipitates were washed multiple times with deionized water and ethanol to remove any residual ions or unreacted precursors. The collected precipitates were then subjected to a drying process before calcination. Drying of the mixture was carried out in an alumina crucible in an oven at 80 °C for 12 h to remove excess moisture and volatile components. This controlled drying step ensured uniform precursor decomposition and minimized agglomeration during calcination. The dried precursor was then ground into fine powder before being calcined at the optimized temperature for phase formation and crystallization. Finally, the powder was calcined at 600 °C for 2 h to remove impurities and unreacted components. The calcined powders were then sintered at 1150 °C for 3 h and ground for 30 minutes. They were then pressed using a homemade hydraulic press at 35 bar cm^−2^. The pellets were circular disks (∼10 mm in diameter and 1–2 mm thick) prepared using polyvinyl alcohol (PVA) as a binder. In the final stage, the compacted powder was re-sintered in air at 1300 °C for 3 hours.

**Table 1:** Chemical formula of the samples and code of Nd_*x*_Co_1−*x*_Fe_2_O_4_

Nd_*x*_Co_1−*x*_Fe_2_O_4_		
*X* = 0	CoFe_2_O_4_	S1
*X* = 0.1	Nd_0.1_Co_0.9_Fe_2_O_4_	S2
*X* = 0.2	Nd_0.2_Co_0.8_Fe_2_O_4_	S3
*X* = 0.3	Nd_0.3_Co_0.7_Fe_2_O_4_	S4
*X* = 0.4	Nd_0.4_Co_0.6_Fe_2_O_4_	S5
*X* = 0.5	Nd_0.5_Co_0.5_Fe_2_O_4_	S6

### 2.3. Instrumental analyses

The crystal structure was examined using a Bruker D8 Advance X-ray diffractometer with CuK_α_ radiation at room temperature. Surface morphology was analyzed *via* Scanning Microscopy (SEM) using a JSM-6360 microscope (JEOL, Japan). To identify the functional groups and vibrational modes of chemical bonds in the prepared ceramics, Fourier-transform infrared spectroscopy (FT-IR) measurement was conducted at room temperature using a Thermo-FT-IR 200 spectrometer (Thermo Scientific, USA).

To measure the dielectric properties, silver paste contacts were made on the sample surface, followed by their annealing at 600 °C for 10 minutes. The frequency-dependent complex dielectric constants (*ε*′, *ε*′′, and tan *δ*), AC conductivity (*σ*_AC_), and complex impedance (*Z*′ and *Z*′′) were all investigated in the frequency range of 1 kHz to 100 kHz, with temperatures ranging from 25 °C to 200 °C, using an LCR meter (TH2826, 20 Hz–5 MHz). Additionally, the room-temperature magnetic properties, including M–H loops, were studied using a vibrating sample magnetometer (VSM7407, Lake Shore).

## 3. Results and discussion

### 3.1. X-ray diffraction

**Fig. 2:**
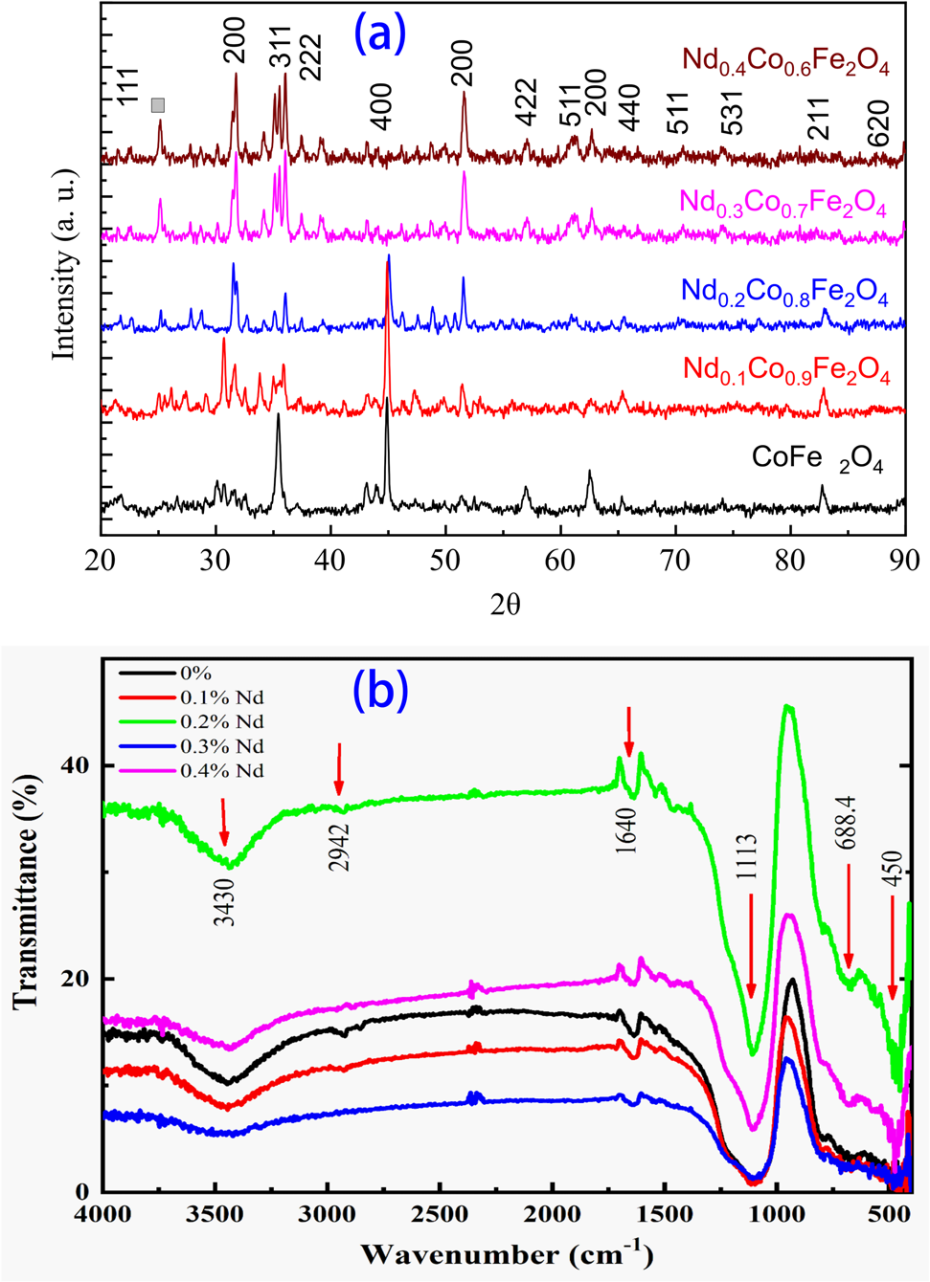
The structural analysis of Nd_(*x*)_Co_(1−*x*)_Fe_2_O_4_: (a) X-ray diffraction and (b) FT-IR transmittance.

The X-ray diffraction pattern of Nd_*x*_Co_1−*x*_Fe_2_O_4_ (0.0 ≤ *x* ≤ 0.5) samples is shown in Fig. 2(a). In the XRD spectrum for all the samples, all the observed characteristic peaks such as (111), (220), (311), (400), (040), (242), (200), (422), (400), (533), and (211) are indexed to the cubic spinel structure of CoFe_2_O_4_ (JCPDS 22-1086).^14^ Additionally, a few small unindexed peaks, which may correspond to a secondary orthorhombic phase of NdFe_2_O_4_, are also observed. The XRD patterns reveal small additional peaks, indicating that the synthesized Nd_*x*_Co_1−*x*_Fe_2_O_4_ samples are in a primary spinel CoFe_2_O_4_ phase, with a secondary phase (NdFe_2_O_4_) also identified. These phases form due to the limited solubility of Nd^3+^ in the spinel lattice, leading to phase segregation at higher doping concentrations. Their presence affects both structural and functional properties. Structurally, they induce lattice strain, altering the crystallite size and microstructure. Functionally, they disrupt Fe^3+^(A)–O–Fe^3+^ (B-site) super-exchange interactions, reducing saturation magnetization (*M*_s_). Additionally, NdFe_2_O_4_ affects electrical resistivity and dielectric properties, influencing energy storage and electronic applications. Controlling these secondary phases is crucial for optimizing material performance in specific technologies. Notably, with increasing Nd^3+^ ion doping, the lattice parameter expanded from 8.3900 to 8.4231 Å, leading to an increase in unit cell volume. Meanwhile, the grain size decreased due to the larger ionic radius of the Nd ion. This is expected because of the large ionic radius of Nd^3+^ in comparison to Fe^3+^, meaning that only a small portion of Fe^3+^ ions may be replaced by Nd^3+^. As a result, Nd^3+^ ions tend to collect near grain boundaries, promoting the development of the NdFe_2_O_4_ phase, as reported in ref. 36.

The phase transformation in Nd-doped CoFe_2_O_4_ can be rationalized by several factors including ionic size effects, crystal field stabilization, and defect chemistry. The ionic radius of Nd^3+^ (0.983 Å) is larger than those of Co^2+^ (0.745 Å) and Fe^3+^ (0.645 Å), which causes lattice distortion upon substitution. Further, the larger Nd^3+^ ions preferentially replace Co^2+^ in A-sites, leading to strain in the spinel structure, which can drive phase transformation into secondary or non-spinel phases. The introduction of Nd^3+^ into CoFe_2_O_4_ affects charge neutrality, possibly requiring the formation of oxygen vacancies to maintain charge balance. Moreover, the presence of oxygen vacancies can destabilize the normal spinel structure and promote the formation of secondary phases such as orthoferrites (NdFeO_3_) or mixed phases (NdCoO_3_). Nd^3+^ has a strong preference for octahedral coordination, whereas Co^2+^ and Fe^3+^ are more flexible in their site occupancy within the spinel lattice. The substitution of Nd^3+^ disrupts the cation distribution in CoFe_2_O_4_, potentially leading to partial inversion of the spinel structure or the emergence of a perovskite-like phase.

At high Nd concentrations, the solubility limit of Nd in the CoFe_2_O_4_ lattice may be exceeded, leading to phase segregation. The Nd–O bond energy is different from those of Co–O and Fe–O, which affects the Gibbs free energy of the system and can drive phase transformation. In addition, the observed phase transformation in Nd-doped CoFe_2_O_4_ likely arises from a combination of lattice distortion, charge compensation mechanisms, and site occupancy changes. The trend aligns with reports on other rare-earth dopants, reinforcing the idea that large trivalent cations in the CoFe_2_O_4_ lattice tend to induce structural transitions due to ionic size mismatch and altered thermodynamic stability. Several studies have reported similar phase transformations in rare-earth-doped CoFe_2_O_4_ systems such as La-doped CoFe_2_O_4_. The La^3+^ substitution also induces lattice distortion and can result in secondary phases such as LaFeO_3_ at high doping levels. In Sm-doped CoFe_2_O_4_, Sm^3+^ doping has been found to reduce the crystallinity of CFO and promote structural disorder, sometimes leading to the appearance of a perovskite phase. In Gd-doped CoFe_2_O_4_, Gd^3+^ doping can induce a transition from a spinel to a mixed spinel-orthoferrite phase, due to strain effects and cation redistribution.^37–39^

The average crystallite size of CoFe_2_O_4_ and Nd-doped CoFe_2_O_4_ ferrite particles was determined using the classical Scherrer's formula.^40^

1 *D*_hkl_ = *kλ*/*β* cos *θ*

where *D*_hkl_ represents the crystallite size calculated from the (311) peak of the XRD profiles, *k* the shape factor (0.89), *θ* is the diffraction angle, *β* is the full width at half-maximum (FWHM) of the peak, and *λ* the wavelength of X-rays (1.54056 Å). The average crystallite sizes of all samples are listed in Table 2. The expansion of the lattice parameter (*a*) is reflected in the increase of the unit cell volume, while the grain size decreases due to the larger ionic radius of the Nd ion.

**Table 2:** X-ray diffraction analysis data for CoFe_2_O_4_ and Nd-doped CoFe_2_O_4_

Samples	Lattice parameter, *a* (Å)	Angles (^o^)	System	Symmetry	Volume (10^6^ pm)^3^	Grain size (nm)	Calculated density (gm cm^−3^)
CoFe_2_O_4_	8.3900	90	Cubic	*Fd*3̄*m*	590.590	11 811.85	5.300
Nd_0.1_Co_0.9_Fe_2_O_4_	8.4010	90	Cubic	*Fd*3̄*m*	592.916	6870.35	5.296
Nd_0.2_Co_0.8_Fe_2_O_4_	8.4140	90	Cubic	*Fd*3̄*m*	595.672	4309.55	5.289
Nd_0.3_Co_0.7_Fe_2_O_4_	8.4170	90	Cubic	*Fd*3̄*m*	596.310	4206.43	5.284
Nd_0.4_Co_0.6_Fe_2_O_4_	8.4211	90	Cubic	*Fd*3̄*m*	597.182	4057.33	5.280
Nd_0.5_Co_0.5_Fe_2_O_4_	8.4231	90	Cubic	*Fd*3̄*m*	597.607	3476.13	5.782

### 3.2. FT-IR analysis

The FT-IR spectra of Nd_*x*_Co_1−*x*_Fe_2_O_4_ (*x* = 0.0, 0.1, 0.2, 0.3, 0.4, and 0.5) samples with wavenumbers ranging from 400 to 3500 cm^−1^ are depicted in Fig. 2(b). All the samples have shown a fundamental absorption peak around 450 cm^−1^, confirming that the Nd_*x*_Co_1−*x*_Fe_2_O_4_ compounds exhibit a cubic spinel ferrite structure. This could be attributed to the stretching vibrations of metal–oxygen bonds.^16,17,24^ Since the atomic mass (A.M.) of Co is greater than that of Fe and the A.M. of O^2−^ remains constant, the overall atomic mass of the metal increases due to the substitution of Fe by Co, as previously reported.^41^ However, the absorption band observed at approximately 688.4 cm^−1^ is attributed to the vibrations of iron–oxygen bonds in the A-site position.^20^ A broad absorption band observed in the range of 2942–3430 cm^−1^ is associated with phase A, which corresponds to the broadening of O–H stretching modes from hydroxyl groups.^18^ In addition, four other absorption bands are also observed that correspond to both (C=O) and C–O vibrations. Both absorption bands observed at 1640 cm^−1^ and 1113 cm^−1^ represent the stretching mode of C=O and C–O vibrations, which in turn arise from esterification reactions.^42,43^

### 3.3. SEM investigation

**Fig. 3:**
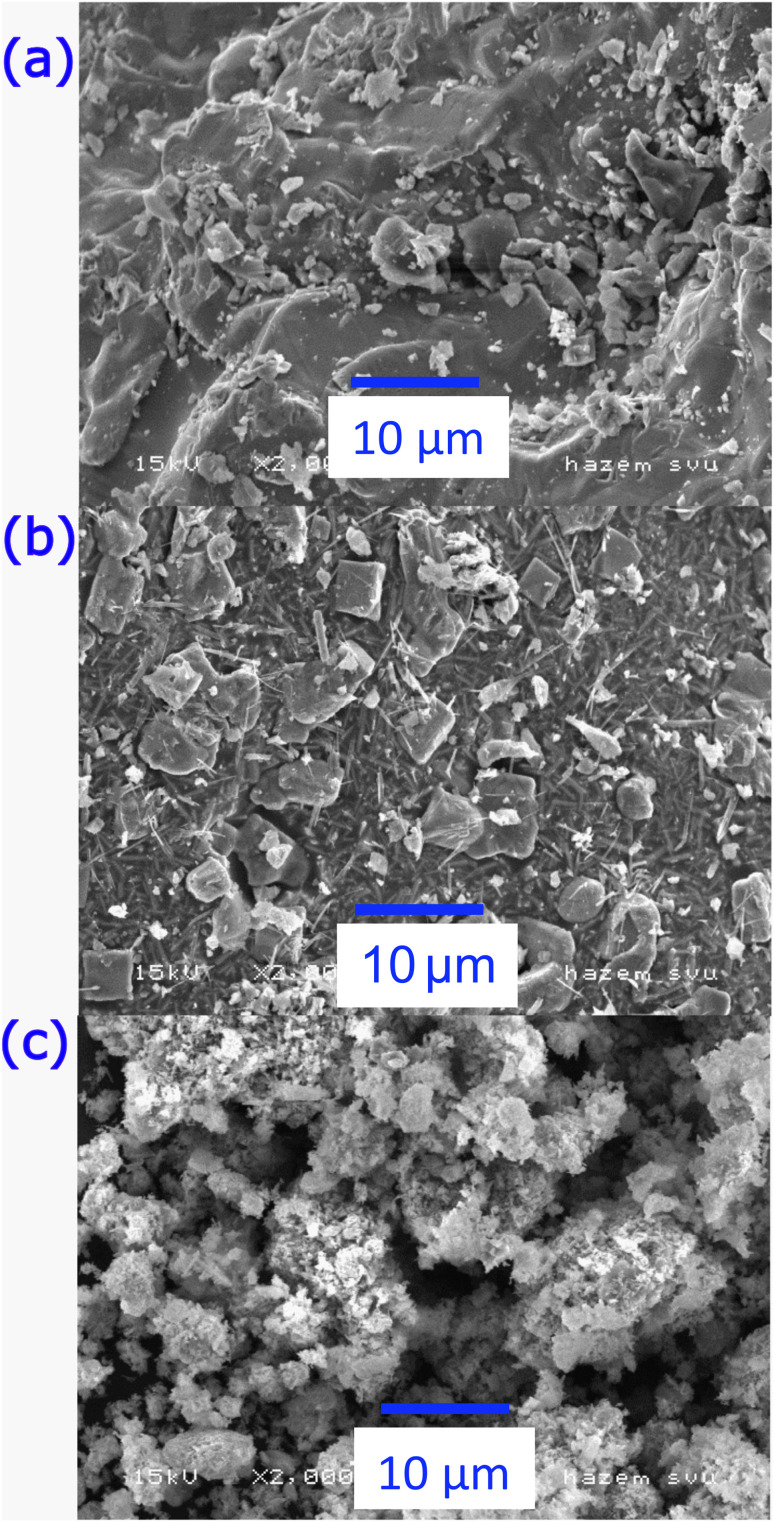
FE-SEM images graph displaying the prepared ferrites' elemental composition and surface morphology: (a) = S2, (b) = S3 and (c) = S4.

To investigate and understand grain size distribution and topology of the grains, FE-SEM micrographs, as shown in Fig. 3(a–c), were analyzed. The FE-SEM micrographs reveal that with the gradual increase of Nd^3+^, the average grain size decreases, making precise statistical calculations challenging. Furthermore, it is evident that the lattice properties have changed when Nd^+^ is substituted in CoFe_2_O_4_ spinel ferrites, which results in an internal stress. Despite this, the Co-ferrite particles have different agglomeration diameters and a hexagonal shape. Here, on the other hand, a more homogeneous distribution of both small and large particles resulted in Nd-doped-ferrite particles exhibiting a roughly cubic structure. Furthermore, because Nd-ferrite grains are larger in ionic size and have a rounder shape than Co-ferrite grains, their surfaces are rougher.

The structural analysis confirmed a cubic spinel ferrite structure in Nd_*x*_Co_1−*x*_Fe_2_O_4_ (0.0 ≤ *x* ≤ 0.5), while at higher Nd concentrations, additional small peaks corresponding to the orthorhombic NdFe_2_O_4_ phase were observed. These peaks are consistent with previous studies on rare-earth doping in spinel ferrites, where excessive Nd substitution leads to the formation of secondary phases due to size and valence mismatch between Nd^3+^ and Fe^3+^ ions.^26^ Nd^3+^ ions have a larger ionic radius (0.983 Å) compared to Fe^3+^ (0.645 Å), which disrupts the spinel lattice and promotes the segregation of an orthorhombic phase at higher doping levels.^44,45^ Similar observations have been reported in Nd-doped cobalt ferrites (Nd_*x*_CoFe_2−*x*_O_4_), where an increase in Nd content beyond a solubility threshold results in the emergence of a secondary rare-earth iron oxide phase.^46^ Additionally, the presence of the orthorhombic NdFe_2_O_4_ phase may be attributed to the stronger Nd–O bond energy, which affects the overall crystallographic stability, as observed in Nd-substituted NiFe_2_O_4_ and ZnFe_2_O_4_ systems.

Moreover, FT-IR spectra further confirm the cubic spinel structure, with characteristic metal–oxygen stretching vibrations. The absorption peaks around *ν* 450 cm^−1^ are attributed to tetrahedral Fe–O stretching, consistent with prior studies on spinel ferrites.^47^ The broad band at 688.4 cm^−1^, corresponding to Fe–O vibrations in the A-site, aligns with previously reported FT-IR studies of Ni-doped ferrites.^48^ The broad O–H stretching modes observed in the 2942–3430 cm^−1^ range indicate the presence of hydroxyl groups, suggesting partial surface hydration or adsorbed moisture, a common phenomenon in ferrite systems.^49,50^ These findings are in agreement with earlier reports on Nd doping in ferrites, where low Nd concentrations stabilize the cubic spinel phase, while higher Nd incorporation induces the segregation of an orthorhombic NdFe_2_O_4_ phase due to ionic radius mismatch and solubility limits.^51^ FT-IR analysis supports the structural interpretation, further validating the formation of the spinel lattice and secondary phases at higher Nd content.

### 3.4. Composition analysis

**Fig. 4:**
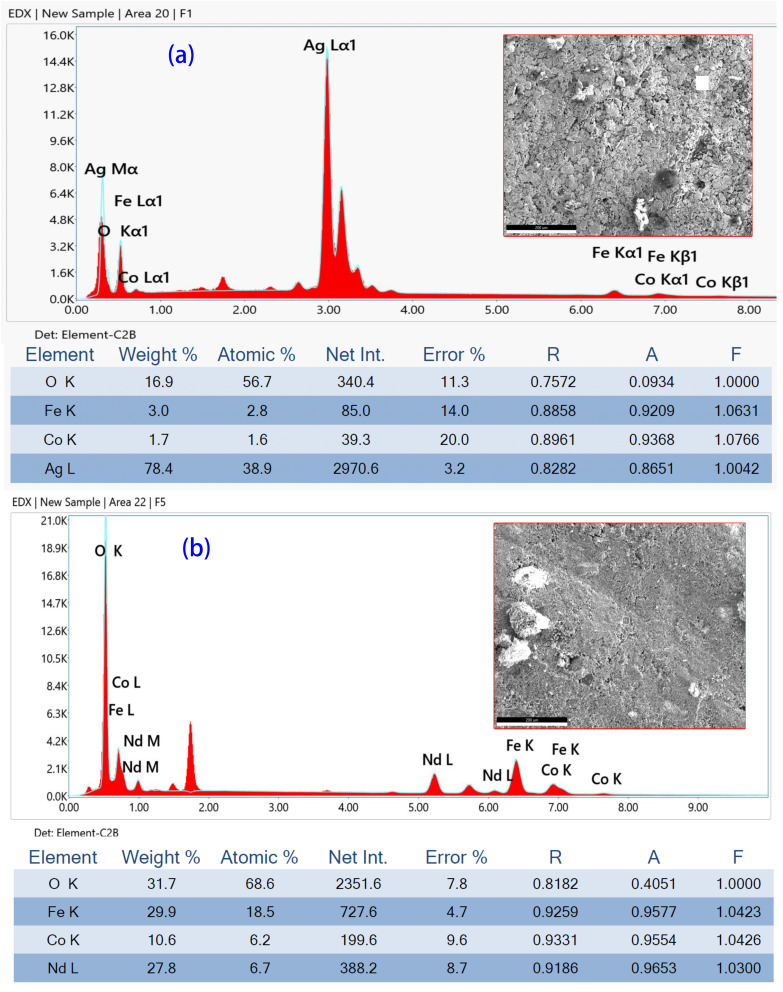
EDX elemental analysis of Nd_*x*_Co_1−*x*_Fe_2_O_4_ (*x* = 0.0 (a) and 0.5 (b)) samples.

Fig. 4 depicts the FE-SEM images and EDS analysis of S1 and S5 samples. To evaluate the elemental composition of the prepared spinel ferrites, EDX analysis was performed on the area shown in FE-SEM images (Fig. 4). The detected elements are all presented in the EDX spectra, from which the proportion of each element was calculated, as shown in the EDX table. The EDX composition table indicates that the weight percentages of each element are nearly within the expected stoichiometric ratios, aligning well with the Nd_*x*_Co_1−*x*_Fe_2_O_4_ (0.0 ≤ *x* ≤ 0.5) composition. All compositions exhibit similar intensity peaks at the same positions, confirming the presence of Nd ions and being consistent with XRD analysis, as shown in Fig. 2. In addition, the EDX spectra show that extremely undesirable precursor materials, like nitrate ions, were successfully removed during the chemical process, and this results in the creation of the required oxide compounds. Here, Nd^3+^ has a larger ionic radius (1.109 Å) compared to Fe^3+^ (0.645 Å) and Co^2+^ (0.745 Å). The incorporation of Nd^3+^ into the CoFe_2_O_4_ spinel structure not only modifies the lattice parameters by inducing expansion due to its larger ionic radius but also influences the overall structural integrity and magnetic characteristics of the material. The preferential occupancy of Nd^3+^ in octahedral sites alters the cation distribution and disrupts the continuity of the crystal lattice. This substitution results in weaker Nd–O bonds compared to the stronger Fe–O interactions, inducing local distortions that can significantly affect crystallite size and increase defect density. Consequently, the presence of Nd^3+^ may impede grain growth, leading to finer microstructures, which in turn can enhance certain functional properties of the spinel ferrites, making them suitable for various applications in magnetic materials and electronics.

### 3.5. Dielectric properties

To study the temperature dependence of dielectric and electrical properties, AC electrical measurements were carried out at 500 kHz within the temperature range of 20 °C to 200 °C. The permittivity (*ε*′) of the samples can be estimated using2
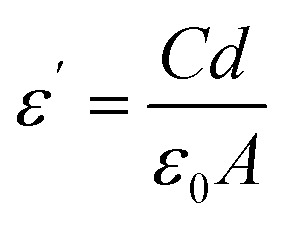
where *A*, *d*, *C*, and *ε*_0_ are the area, thickness, capacitance, and dielectric constant of free space (8.854 × 10^−12^ F m^−1^), respectively. The dielectric tangent loss (tan(*δ*)) can be determined using the equation

3 *D* = tan(*δ*) = 1/(*ωR*_p_*C*_p_).

To calculate AC conductivity (*σ*_ac_), the following relationship is employed:

4 *σ*_ac_ = *ωε*_o_*ε*_r_ tan(*δ*).

5 *ω* = 2π*f*

**Fig. 5:**
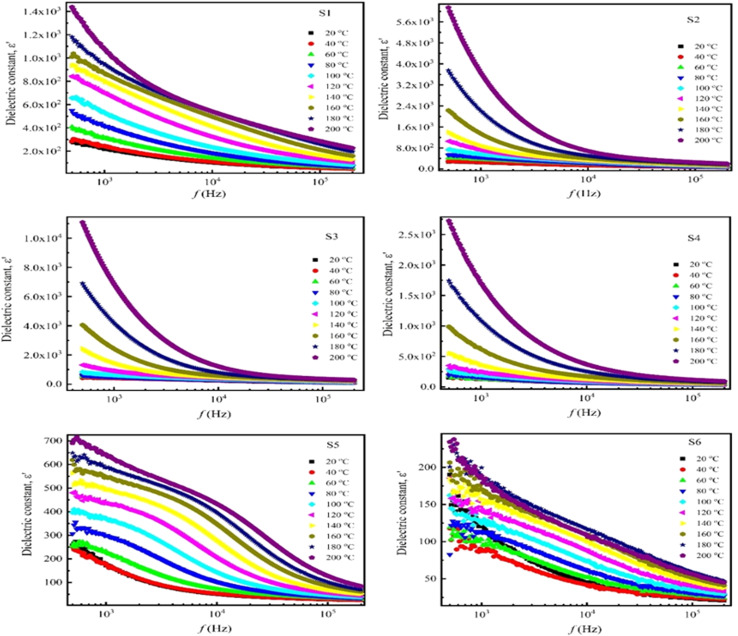
Dielectric constant (*ε*′) as a function of frequency in the temperature range (25–200 °C) of Nd_*x*_Co_1−*x*_Fe_2_O_4_ (*x* = 0–0.5).

The frequency dependence of the dielectric constant (permittivity) of Nd_*x*_Co_1−*x*_Fe_2_O_4_ (*x* = 0–0.5) in the frequency range (100 Hz–1 MHz) with varying temperatures (25 °C to 200 °C) is shown in Fig. 5. Interestingly, all samples showed a substantially high dielectric constant in the low-frequency band, with the dielectric constant dropping exponentially with increasing frequency. This is caused by the frequency-varying effects of ionic, dipolar, electronic, and space charge polarizations. However, eventually, the dielectric constant stabilizes at high frequencies, signifying that the material's polarization has reached saturation. Thereafter, there is no discernible change in the dielectric constant with increasing frequency, indicating the dominance of two different polarization mechanisms.^52^ This frequency-independent behavior is commonly observed in most ferrimagnetic materials and ascribed to the transfer of Fe^3+^ ions into Fe^2+^ ions during the sintering process. A dipolar ferrite material is usually produced when there are more Fe^3+^ ions than Fe^2+^ ions. Polarization relaxation occurs in ferrites when the coupling of Fe^3+^ and Fe^2+^ ions aligns with an alternating field.^53^ The decrease in the dielectric constant can be ascribed to space charge carriers,^54^ as it requires a certain time interval for orientation in response to the applied field. As the frequency increases, these space charge carriers lag behind the changing field, resulting in a reduced dielectric constant.^55^ Conversely, the dielectric constant, which measures permittivity, increases with temperature for all samples, reaching a maximum during the transition from ferroelectric to paraelectric. This increase may be due to space charge polarization, as higher temperature enhances the interfacial polarization, which in turn increases permittivity. However, with the application of an electric field on the dielectric materials, the polarization implies the harvesting charges, and the effects of temperature on ionic and electronic polarization are a minimal.^35^ Additionally, because of the different ionic radii of Nd^3+^ (1.109 Å) and Co^2+^ (0.745 Å), it seems the Nd^3+^ ions preferred the A-site position, causing a threefold increase in the dielectric constant with increasing Nd doping.^56^

**Fig. 6:**
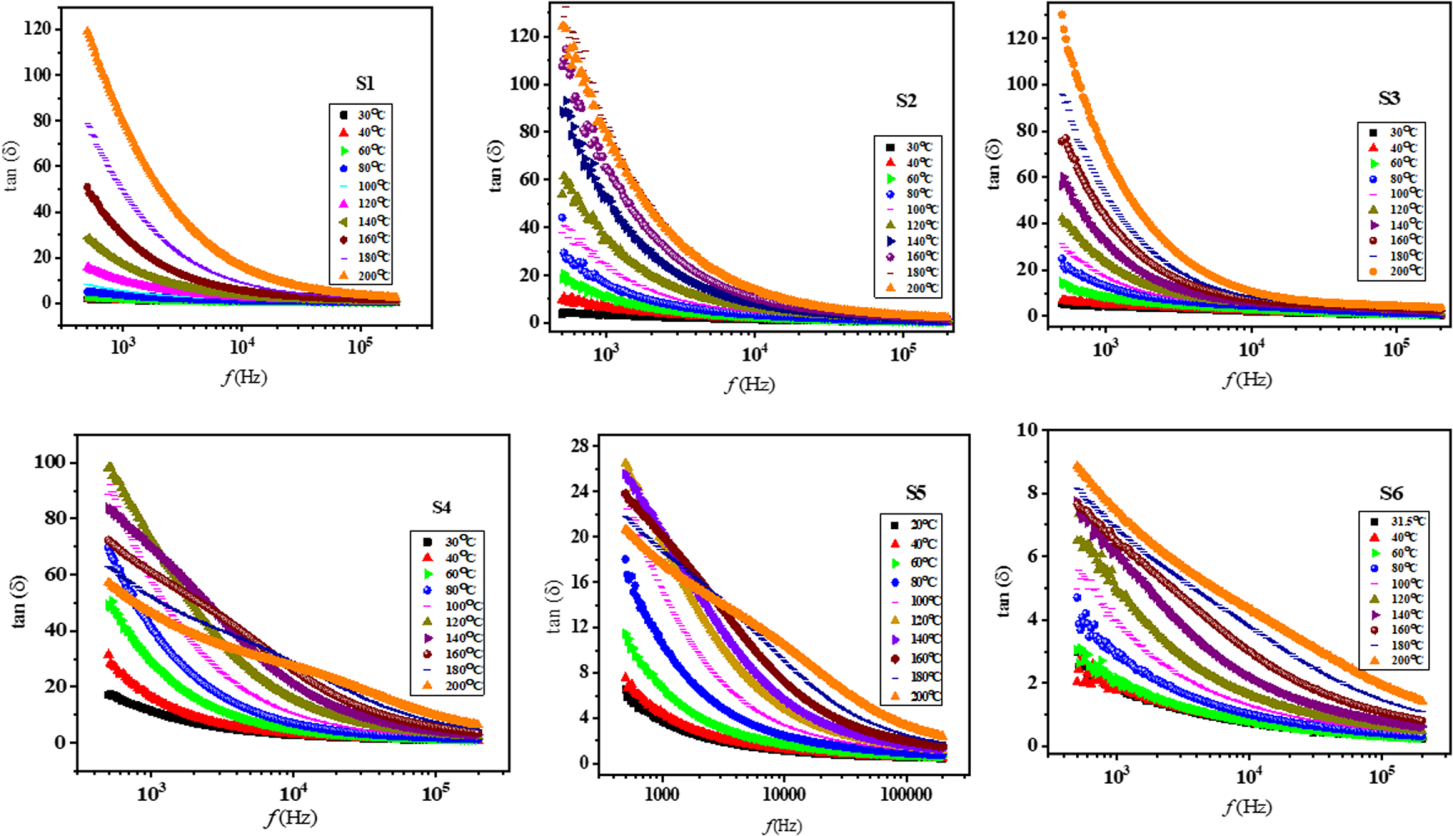
Frequency-dependent dielectric loss tangent (tan *δ*) at altered temperatures ranging from 25 °C to 200 °C for Nd_*x*_Co_1−*x*_Fe_2_O_4_ (*x* = 0–0.5).

Fig. 6 shows the dielectric loss tangent (tan(*δ*)) as a function of frequency for all Nd_*x*_Co_1−*x*_Fe_2_O_4_ (*x* = 0–0.5) samples at different temperatures (30 to 200 °C). The dielectric loss tangent for all samples exhibits a similar trend to that of *ε*′. At low frequency, tan *δ* is higher and decreases exponentially as frequency increases. This occurs because, at low frequencies, the applied field frequency is lower than the electron hopping frequency between Fe^2+^ and Fe^3+^ ions at the B-site, thus leading to greater dielectric loss. Conversely, at high frequencies, the dielectric loss becomes constant under the applied electric field. As the frequency improves, both space charge polarization and dielectric loss decrease because the electron hopping between Fe^2+^ and Fe^3+^ ions can no longer keep up with the applied field.^57^ Most of the energy loss at low frequencies was recognized due to huge resistant grain boundaries. Therefore, energy loss is higher at low frequencies and lower at high frequencies. This is because, at low frequencies, more energy is essential for electron hopping between ferrous (Fe^2+^) and ferric (Fe^3+^) ions, compared to the energy required at higher frequencies.^58–60^ Additionally, the temperature dependence of dielectric loss reveals that tan *δ* rises as the temperature increases. This behavior is most likely because of exchange between Fe ions in octahedral positions, which is influenced by the movement of charge carriers.^61^ Moreover, the dielectric loss tangent is minimized for doped samples *x* ≈ 0.1 and *x* ≈ 0.5.

### 3.6. AC conductivity

**Fig. 7:**
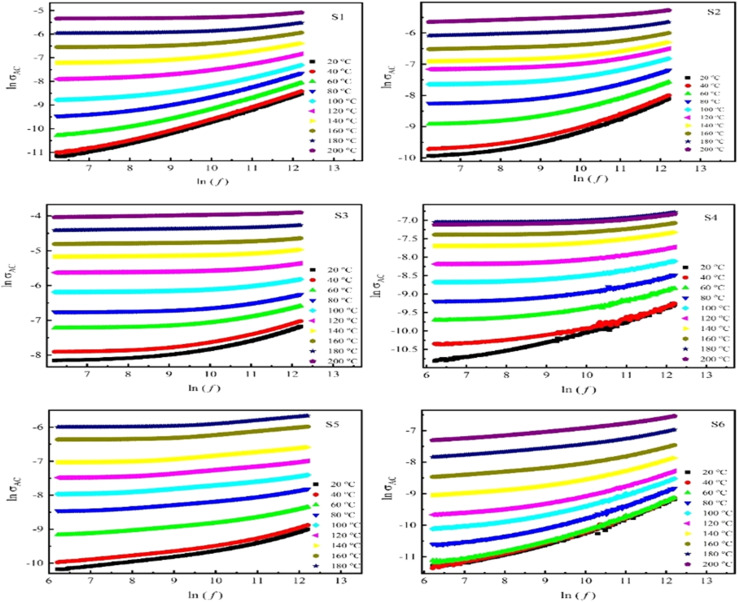
AC conductivity through variation of ln(*σ*_ac_) dependence ln(*f*) plots with temperatures (20–200 °C) of all samples.

The AC technique distinguishes between various mechanisms that contribute to the material's overall conductivity response, like electrode response and the electrical conduction through grains and their boundaries. However, the DC technique reflects the total conductivity response of the material. Fig. 7 presents the ln(*σ*_ac_) *versus* ln(*f*) plots with temperatures. The analysis reveals that all samples exhibit a nearly identical behavior with two distinct slopes, implying a rise in conductivity with rising temperature. The slopes at higher frequencies are steeper than those at lower frequencies, as high-frequency radiation exerts a greater driving force on charge carriers, resulting in higher AC conductivity at elevated frequencies.^62^ According to *Koops* model, ferrite samples behave like multilayer capacitors which are combined grain with boundary,^63^ resulting in the increase in the conductivity at higher frequency and elevated temperature. At lower frequencies, a nearly continuous plateau is noted, where electronic charge carriers are hindered from hopping between the more resistive grain boundaries. However, with increasing frequencies, the conductive grains were active, enabling charge carriers hopping between neighboring ions.^64^ In DC conductivity, charge carriers follow the shortest path between ions, which includes jumps across regions with higher resistance, such as the spaces between cations, a factor that is not required in AC conduction. As a result, AC conduction may involve lower activation energy.^65–67^

### 3.7. Dielectric-temperature dependence

**Fig. 8:**
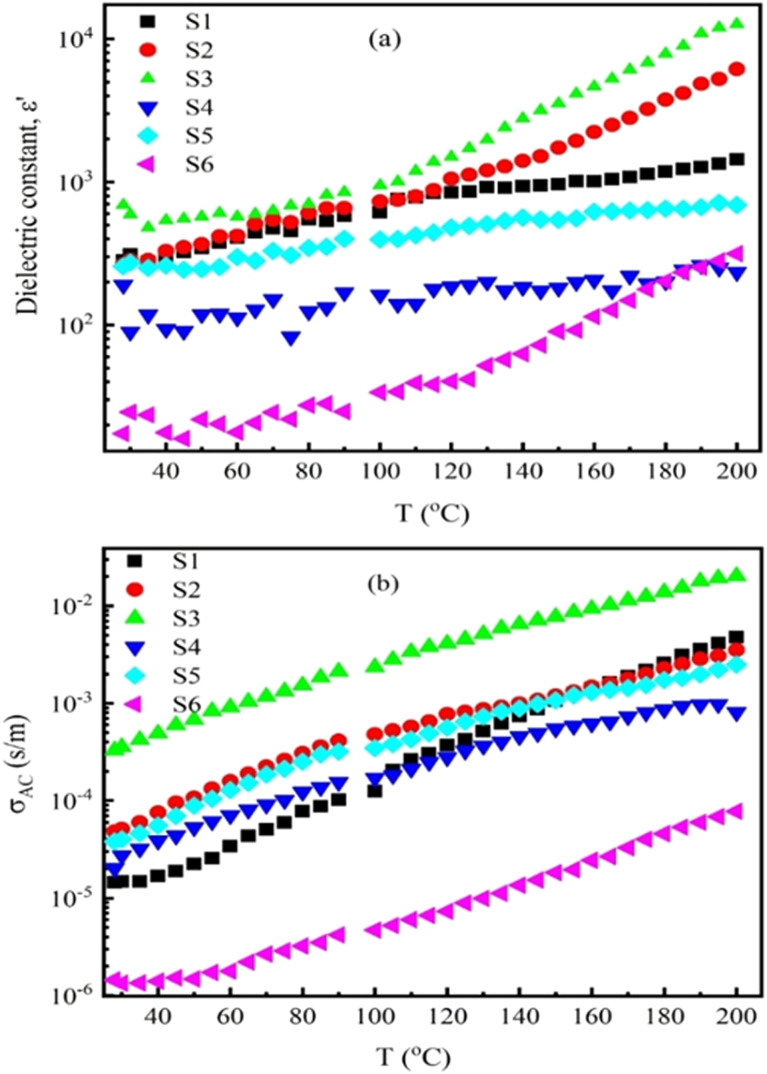
Temperature-dependent ac conductivity of Nd^3+^ doped cobalt-ferrite at different temperatures.

Fig. 8(a) illustrates the dielectric constant (*ε*′) of Nd_*x*_Co_1−*x*_Fe_2_O_4_ as a function of temperature for all samples at an applied frequency of 500 kHz. It is observed that *ε*′ increases monotonically with rising temperature. However, at higher temperatures, the dielectric constant remains nearly constant. This is because the dipoles acquire sufficient thermal energy to align with the applied field, resulting in higher dielectric constant values. In contrast, at lower temperatures, there is insufficient energy to fully polarize the dipoles.^68^

Furthermore, with the increased doping concentration of Nd^3+^ ions, the dielectric constant decreases as well.^69^ This is ascribed to the large ionic radius of Nd^3+^, which preferentially occupies octahedral B-sites, replacing Fe^3+^ ions. Consequently, Nd^3+^ doping in Co-ferrites forces Fe^3+^ to move its position from B to A sites, and in turns it can reduce the Fe^3+^ number in the B-site, leading to decreased interstitial polarization.^70,71^ As a result, the dielectric constantly reduces.

Fig. 8(b) shows the *σ*_ac_*versus* frequency plot for all Nd_*x*_Co_1−*x*_Fe_2_O_4_ (*x* = 0.0 to 0.5) composites with varying temperature (25–200 °C) at a constant frequency of 500 kHz. The temperature-dependent AC conductivity measurements show that *σ*_ac_ gradually increases with the increase of temperature for all samples. Specifically, in the low-temperature region (*T* < 100 °C), *σ*_ac_ increases slightly. However, it becomes more significant when the temperature increases (*T* > 100 °C). This is due to the increased applied field, which enhances the electron mobility and hence leads to higher conductivity values.

The behavior of AC conductivity with varying frequency was described using a large/small polaron model. This concept states that when an electric field is applied, electrons begin to migrate and polarize the surrounding lattice by creating polarons. Massive polarons are produced when the distortion increases the lattice constant; tiny polarons appear when the distortion is equal to the lattice constant.^72,73^

### 3.8. Impedance spectroscopy

The real (*Z*′) and imaginary (*Z*′′) parts of the impedance were calculated for Nd_*x*_Co_1−*x*_Fe_2_O_4_ using the following equation:^74^

6 *Z*′ = *Z* cos *θ*

7 *Z*′′ = *Z* sin *θ*

*Z* is the complex impedance and *θ* represents the phase angle. *Z*′ and *Z*′′ correspond to the real and imaginary parts of the measured impedance, respectively.

#### 3.8.1. Real impedance (*Z*′) with various frequencies and temperatures

**Fig. 9:**
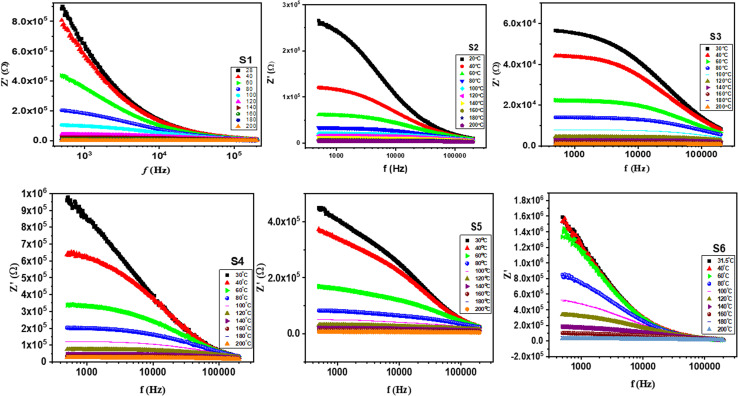
Real impedance (*Z*′) as a function of frequency for S1, S2, S3, S4, S5 and S6 from room temperature to 200 °C.

**Fig. 10:**
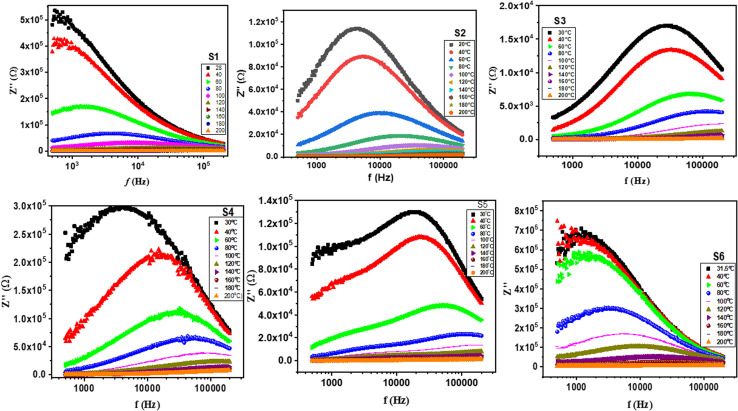
Imaginary impedance (*Z*′′) as a function of frequency for S1, S2, S3, S4, S5 and S6 from RT to 200 °C.

Fig. 9 illustrates the real part of impedance (*Z*′) at various frequencies and temperatures. The real fraction of the *Z*′ value decreases when the field is applied more intensely.^46^ The *Z*′ value converges and becomes temperature independent at high frequencies. This phenomenon is attributed to space charge polarization, which stops charge accumulation at interfaces, boosting conductivity and stopping the process of space charge polarization.^75^Fig. 10 depicts the imaginary part of impedance (*Z*′′) with frequency at varying temperatures. The *Z*′′ value first increases with increasing frequency, reaching a maximum value (*Z*′′ max), and subsequently declines, indicating the existence of an electrical relaxation phenomenon.^76^

#### 3.8.2. Cole–Cole plots

**Fig. 11:**
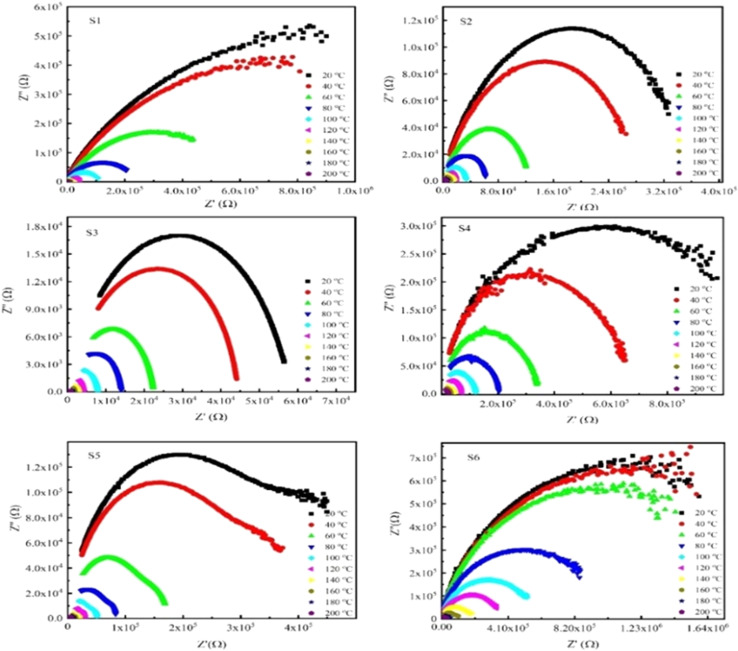
The *Z*′ and *Z*′′ as a function of temperature (30 to 200 °C) for Nd_*x*_Co_1−*x*_Fe_2_O_4_ (*x* = 0–0.5).

The Cole–Cole plots for all samples at temperatures ranging from 30 to 200 °C are presented in Fig. 11. Plots at lower temperatures (30, 40, and 60 °C) display a semicircular arc, indicating that the bulk material does not contribute to conduction, which mostly happens at grain boundaries and the electrode contacts. It also suggests the existence of a single relaxing process.^61,62,77^ The capacitive behavior of the material is shown in the noticeable shift in the arc caused by the resistance of the grain boundaries and electrode interface decreasing with increasing temperature.^78^ The single semicircular arc observed for both pure and Nd-doped samples further emphasizes the importance of intergranular boundaries in determining impedance. Furthermore, the radius of a semicircle reduces as temperature rises, indicating decreased resistance and improved conduction at higher temperatures.^64,79^ These findings suggest that the choice of the ferrite dopant and doping level can significantly affect resistance and resistivity, opening new avenues for optimizing ferrite materials for desired applications.

### 3.9. Magnetic properties (M–H hysteresis loops)

**Fig. 12:**
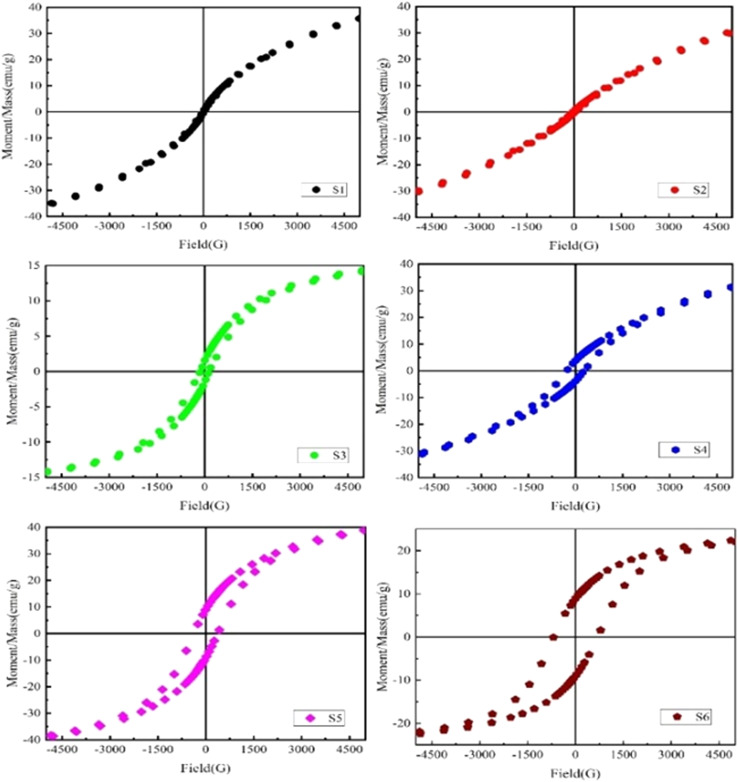
M–H hysteresis loops of Nd_*x*_Co_1−*x*_Fe_2_O_4_ (*X* = 0–0.5) for all investigated samples.

The magnetic hysteresis loops (M–H) of the manufactured Nd_*x*_Co_1−*x*_Fe_2_O_4_ solid solutions (*x* = 0–0.5) were measured using VSM under an applied field of ±40 kOe, as shown in Fig. 12. The M–H loops reveal the effect of the Nd ions on the magnetic properties including coercivity (*H*_c_), remnant magnetization (*M*_r_), and saturation magnetization (*M*_s_). All the samples showed a narrow, “S”-shaped M–H curve, being the characteristic of the ferromagnetic state. The squareness ratio (*M*_r_/*M*_s_) increases with increasing Nd doping. Typically, in cobalt ferrite (CoFe_2_O_4_), Co^2+^ ions occupy the octahedral (B-site) positions, while Fe^3+^ ions reside in both tetrahedral (A) and octahedral (B) sites.^80,81^ However, with the doping of Nd^3+^ ions, it prefers the tetragonal structure at B-sites, causing migration of Co^2+^ and Fe^3+^ ions from B-sites to A-sites, thereby leading to an increased squareness (*M*_r_/*M*_s_) ratio.

The saturation magnetization (*M*_s_) and retentivity (*M*_r_) both increase with rising Nd^3+^ concentrations up to *x* = 0.1 and 0.3, after which they begin to decrease, as depicted in Fig. 12. For the sample with 2% Nd doping, *M*_s_ is approximately 3.08 emu g^−1^ and *H*_c_ is around 64.26 Oe. For the 4% doped sample, these values rose to 5.621 emu g^−1^ and 143.43 Oe, correspondingly. Owing to the larger magnetic moment of Nd^3+^ (3.65 μB) in the position of a lower magnetic moment of Co^2+^ (3 μB) at B-sites, the saturation magnetization increases. However, the increased coercivity can be related to the existence of the NdFe_2_O_4_ phase of the parent compound with the primary phase, causing significant lattice distortion.^82^ Notably, our findings show higher magnetization as compared to previous reports about Nd-doped BFO synthesized *via* the sol–gel method.^83,84^ This strong saturation magnetization at room temperature (300 K) is linked to the variations in chemical composition and cation distribution, as suggested by the Néel sub-lattices model.^85^ These findings offer promising potential for applications in memory devices and spintronics. The physical traits of Nd-doped CoFe_2_O_4_, including particle size, morphology, density, porosity, and cation distribution, significantly influence its magnetic properties. Larger particles enhance magnetization (*M*_s_) through stronger exchange interactions, while smaller particles increase coercivity (*H*_c_) due to single-domain behavior but may induce superparamagnetic at extreme sizes. Morphology affects anisotropy, with elongated structures increasing *H*_c_. A higher density improves exchange coupling, increasing *M*_s_ and decreasing *H*_c_, whereas porosity weakens interactions, reducing *M*_s_ while increasing *H*_c_*via* domain wall pinning. Nd^3+^ doping alters cation distribution, reducing *M*_s_ and modifying *H*_c_, with excessive doping leading to secondary phase formation. For high-frequency applications, controlled particle size and porosity minimize eddy current losses, while Nd-induced anisotropy enhances ferromagnetic resonance, making Nd-doped CoFe_2_O_4_ suitable for transformers, sensors, and spintronic devices.^25^

## 4. Conclusions

Spinel ferrites doped with the rare-earth element Nd^3+^, forming the solid solution Nd_*x*_Co_1−*x*_Fe_2_O_4_ (*x*; 0.0–0.5), were effectively synthesized using the sol–gel technique. XRD analysis confirmed the cubic phase spinel structure of CoFe_2_O_4_. The Nd doping significantly influenced the dielectric properties of the prepared materials, which was ascribed to the reduction of Co^2+^ ions and the incorporation of Nd^3+^ ions. FT-IR analysis confirms the cubic spinel ferrite structure of Nd_*x*_Co_1−*x*_Fe_2_O_4_, with characteristic metal–oxygen stretching vibrations. AC conductivity as a function of frequency was explained using the large/small polaron model. The impedance and electrical modulus show a Debye-type relaxation phenomenon. Cole–Cole plots exhibited semicircular arcs at 30, 40, and 60 °C, which indicates that the conduction mechanism in Nd-doped CoFe_2_O_4_ is governed by grain boundary effects and space charge polarization, supporting the polaron hopping conduction mechanism. Further, Nd-doped CoFe_2_O_4_ exhibits enhanced magnetic properties with increasing Nd^3+^ concentration, reaching 5.621 emu g^−1^ (*M*_s_) and 143.43 Oe (*H*_c_) for the 0.4 doped sample. Strong saturation magnetization at 300 K suggests a key role of chemical composition and cation distribution, as explained by the Néel sub-lattice model. Compared to Nd-doped BFO, our samples show superior magnetic performance. A transition from an antiferromagnetic to a ferromagnetic state was observed, with a high Curie temperature (*T*_m_) of 292 °C, confirming a stable ferromagnetic phase, making Nd-doped CoFe_2_O_4_ promising for permanent magnet applications.

## Ethical approval

No ethical approval was granted to conduct the experiments involved within the manuscript.

## Data availability

The data sets used and/or analyzed during the current study are available from the corresponding author on reasonable request.

## Author contributions

Amr Ali and Ahmed I. Ali: formal analyses, experiments and data analysis; Ahmed I. Ali: project administration, resources, and writing – the original draft; Galal H. Ramzy: data analysis and software; Muhammad Arif, Nasser Zouli, Ahmed F. F. Abouatiaa, and Abdel Samed M. Adam: wrote – the original draft of the manuscript; and Saleh M. Matar, Ahmed I. Ali and Elbadawy A. Kamoun: supervision, software, and wrote – reviewed and editing the final draft. All authors approved the current version of the draft for submission.

## Conflicts of interest

The authors declare that they have no known competing financial interests or personal relationships that could have appeared to influence the work reported in this paper.

## References

[cit1] Houshiar M. (2014). *et al.*, Synthesis of cobalt ferrite (CoFe2O4) nanoparticles using combustion, coprecipitation, and precipitation methods: A comparison study of size, structural, and magnetic properties. J. Magn. Magn. Mater..

[cit2] Zi Z. (2009). *et al.*, Synthesis and magnetic properties of CoFe2O4 ferrite nanoparticles. J. Magn. Magn. Mater..

[cit3] Yadav R. S. (2016). *et al.*, Impact of Nd3+ in CoFe2O4 spinel ferrite nanoparticles on cation distribution, structural and magnetic properties. J. Magn. Magn Mater..

[cit4] Giri A. (2002). *et al.*, Photomagnetism and structure in cobalt ferrite nanoparticles. Appl. Phys. Lett..

[cit5] Arulmurugan R. (2006). *et al.*, Mn–Zn ferrite nanoparticles for ferrofluid preparation: Study on thermal–magnetic properties. J. Magn. Magn Mater..

[cit6] Jauhar S. (2016). *et al.*, Tuning the properties of cobalt ferrite: a road towards diverse applications. RSC Adv..

[cit7] Chandekar K. V., Shkir M., AlFaify S. (2020). A structural, elastic, mechanical, spectroscopic, thermodynamic, and magnetic properties of polymer coated CoFe2O4 nanostructures for various applications. J. Mol. Struct..

[cit8] Melo R., Banerjee P., Franco A. (2018). Hydrothermal synthesis of nickel doped cobalt ferrite nanoparticles: optical and magnetic properties. J. Mater. Sci.: Mater. Electron..

[cit9] Ammar S., Fiévet F. (2020). Polyol synthesis: A versatile wet-chemistry route for the design and production of functional inorganic nanoparticles. Nanomaterials.

[cit10] Pinheiro A. (2020). *et al.*, Exchange bias and superspin glass behavior in nanostructured CoFe2O4-Ag composites. J. Magn. Magn. Mater..

[cit11] Rajendran M. (2001). *et al.*, Magnetic properties of nanocrystalline CoFe2O4 powders prepared at room temperature: variation with crystallite size. J. Magn. Magn. Mater..

[cit12] Edelman I. (2019). *et al.*, Effect of gadolinium on magnetic circular dichroism and electron magnetic resonance of ε-Fe2O3 nanoparticles formed in borate glasses. J. Non-Cryst. Solids.

[cit13] Kurian M. (2015). *et al.*, Structural, magnetic, and acidic properties of cobalt ferrite nanoparticles synthesised by wet chemical methods. J. Adv. Ceram..

[cit14] Fkhar L. (2019). *et al.*, Magnetic and structural properties of novel neodymium-tin spinel ferrite nanoparticles. J. Supercond. Novel Magn..

[cit15] Pandiyan R. , Growth by Radio Frequency Sputtering and Characterisation of Rare Earth Doped Wide Bandgap Oxides, University of Trento, 2013

[cit16] Jalaiah K. (2018). *et al.*, Co-dopant affect on the structural, electrical and magnetic properties of zirconium and copper co-substituted Ni0. 75Zn0. 25Fe2O4 spinel ferrites synthesized by sol-gel method. Chin. J. Phys..

[cit17] Şabikoğlu İ. (2015). *et al.*, The effect of neodymium substitution on the structural and magnetic properties of nickel ferrite. Prog. Nat. Sci.: Mater. Int..

[cit18] Ahmadpour G. (2021). *et al.*, Microstructure, composition and magnetic properties of Nd-(Fe1-xCox)-B oxide magnetic particles synthesized by Pechini-type chemical method. Adv. Powder Technol..

[cit19] Hunyek A., Sirisathitkul C., Harding P. (2010). Synthesis and characterization of CoFe2O4 particle by PVA sol-gel method. Adv. Mater. Res..

[cit20] Mahhouti Z. (2019). *et al.*, Chemical synthesis and magnetic properties of monodisperse cobalt ferrite nanoparticles. J. Mater. Sci.: Mater. Electron..

[cit21] Kumar N. H. (2023). *et al.*, Structural, optical, thermoelectric, and magnetic properties of terbium doping on zinc cobalt nanoparticles and applications. Indian J. Phys..

[cit22] Ahmed M., Okasha N., Gabal M. (2004). Transport and magnetic properties of Co–Zn–La ferrite. Mater. Chem. Phys..

[cit23] Ahmed M., Okasha N., Ebrahem A. (2005). Correlation of the physico chemical properties of Zn-substituted Li–La ferrite. Ceram. Int..

[cit24] Kharazi P., Rahimi R., Rabbani M. (2019). Copper ferrite-polyaniline nanocomposite: structural, thermal, magnetic and dye adsorption
properties. Solid State Sci..

[cit25] Azam F. , Ali H. and Anis-ur-Rehman M., Investigation of Structural, Electrical, and Resistive Switching Effect of Co-Gd Ferrites by Substituting Nd+3 for Rram Applications, SSRN: https://ssrn.com/abstract=5129776

[cit26] Almessiere M. (2024). *et al.*, Exploring dielectric and electrical characteristics in Sr0. 5Ba0. 5SnxFe12-xO19/CoFe2O4 nanocomposites. Ceram. Int..

[cit27] Ghadi F. R., Hodtani G. A. (2020). Copula-based analysis of physical layer security performances over correlated Rayleigh fading channels. IEEE Trans. Inf. Forensics Secur..

[cit28] Anil P. (2025). *et al.*, Structural and dielectric characteristics of CoFe2O4/nitrogen-enriched reduced graphene oxide composites via a one-pot solvothermal method. J. Mater. Sci..

[cit29] Srinivasamurthy K. (2025). *et al.*, Electrochemical performance of Sr-doped cobalt nickel ferrite ceramics for supercapacitor applications. J. Energy Storage.

[cit30] Du X., Graedel T. E. (2011). Global rare earth in-use stocks in NdFeB permanent magnets. J. Ind. Ecol..

[cit31] Zhang Y. (2020). *et al.*, Hydrometallurgical recovery of rare earth elements from NdFeB permanent magnet scrap: A review. Metals.

[cit32] Kalyankar S. S. (2024). *et al.*, Composite Materials For Adsorption of Rare Earth Metal Ions. Water, Air, Soil Pollut..

[cit33] Firdaus M. (2016). *et al.*, Review of high-temperature recovery of rare earth (Nd/Dy) from magnet waste. J. Sustain. Metall..

[cit34] Wang H., Lamichhane T. N., Paranthaman M. P. (2022). Review of additive manufacturing of permanent magnets for electrical machines: A prospective on wind turbine. Mater. Today Phys..

[cit35] Vani K. (2025). *et al.*, Impact of rare earth Tb3+ substitution in cobalt ferrites: Tuning structural, dielectric, magnetic properties and photocatalytic activity. Ceram. Int..

[cit36] Bercoff P. G., Herme C., Jacobo S. E. (2009). The influence of Nd–Co substitution on the magnetic properties of non-stoichiometric strontium hexaferrite nanoparticles. J. Magn. Magn. Mater..

[cit37] Yadav R. S. (2015). *et al.*, Structural and Magnetic Properties of CoFe2− xGdxO4 (0.0≤ x ≥ 0.1) Spinel Ferrite Nanoparticles Synthesized by Starch-Assisted Sol–Gel Auto-combustion Method. J. Supercond. Novel Magn..

[cit38] Song W. (2024). *et al.*, Enhancing the performance of spinel La-doped CoFe2O4 oxygen carriers for chemical looping hydrogen generation. Green Carbon.

[cit39] Amar I. A. A. , New materials for electrochemical synthesis of ammonia, Doctoral Thesis, Department of Pure and Applied Chemistry, University of Strathclyde, 2014

[cit40] Patterson A. (1939). The Scherrer formula for X-ray particle size determination. Phys. Rev..

[cit41] Evangelou V. , Pyrite Oxidation and its Control, CRC press, 2018

[cit42] Vivekanandhan S., Venkateswarlu M., Satyanarayana N. (2005). Effect of different ethylene glycol precursors on the Pechini process for the synthesis of nano-crystalline LiNi {sub 0.5} Co {sub 0.5} VO {sub 4} powders. Mater. Chem. Phys..

[cit43] Vivekanandhan S., Venkateswarlu M., Satyanarayana N. (2005). Effect of different ethylene glycol precursors on the Pechini process for the synthesis of nano-crystalline LiNi0. 5Co0. 5VO4 powders. Mater. Chem. Phys..

[cit44] Song X.-L. (2020). *et al.*, NdFe2O4 Nanoparticles: Synthesis, Characterization, and Magnetic Properties. Sci. Adv. Mater..

[cit45] Song X. (2023). *et al.*, Study on the controllable preparation of Nd3+ doped in Fe3O4 nanoparticles for magnetic protective fabrics. Molecules.

[cit46] Moreno-Góngora A. , Sonochemical Synthesis of Ferrite Nanoparticles, 2023

[cit47] Dawoud H., Ouda L., Shaat S. (2017). FT-IR studies of nickel substituted polycrystalline zinc spinel ferrites for structural and vibrational investigations. Chem. Sci. Trans..

[cit48] Yadav R. S. (2017). *et al.*, Structural, magnetic, dielectric, and electrical properties of NiFe2O4 spinel ferrite nanoparticles prepared by honey-mediated sol-gel combustion. J. Phys. Chem. Solids.

[cit49] Petrila I., Tudorache F. (2022). Annealing temperature effects on humidity sensor properties for Mg0. 5W0. 5Fe2O4 spinel ferrite. Sensors.

[cit50] Mathpal M. C. , *et al.*, State of art of spinel ferrites enabled humidity sensors, Spinel Nanoferrites: Synthesis, Properties and Applications, 2021, pp. 437–475

[cit51] Saha M. (2022). *et al.*, Structural, optical, dielectric, and magnetic properties of spinel MFe2O4 (M= Co and Zn) nanoparticles synthesized by CTAB-assisted hydrothermal method. Ceram. Int..

[cit52] Abdelwahab S. A. (2021). *et al.*, Influence of TiO2/GO weight ratio on the structure, mechanical, and electrical properties of SiO2–Al2O3 glass–ceramics. J. Mater. Sci.: Mater. Electron..

[cit53] Murad E. and Cashion J., Mössbauer Spectroscopy of Environmental Materials and Their Industrial Utilization, Springer Science & Business Media, 2011

[cit54] Torabi S. (2015). *et al.*, Strategy for enhancing the dielectric constant of organic semiconductors without sacrificing charge carrier mobility and solubility. Adv. Funct. Mater..

[cit55] Sajedi Alvar M., Blom P. W., Wetzelaer G.-J. A. (2020). Space-charge-limited electron and hole currents in hybrid organic-inorganic perovskites. Nat. Commun..

[cit56] Zannen M. (2012). *et al.*, Structural, optical, and electrical properties of Nd-doped Na0. 5Bi0. 5TiO3. Mater. Chem. Phys..

[cit57] Ranga R. (2025). *et al.*, Effect of La3+ ion doping on Structural, Magnetic and Dielectric behaviour of Mg0. 5Co0. 5Fe2O4 (0≤ x≤ 0.1). Phys. Chem. Chem. Phys..

[cit58] Alanazi Y. (2024). *et al.*, Improved structural and dielectric traits of Cd-Zn spinel ferrites by co-substitution of La 3+ and Er 3+ cations. J. Ovonic Res..

[cit59] Ali A. I. (2023). *et al.*, Dielectric and dynamic antibacterial investigations of organic–inorganic conductive membranes based on oxidized cellulose with BNKT nanoceramics. Cellulose.

[cit60] Chikhale R. N., Shinde V. S., Bhatia P. G. (2024). Investigate structural, morphological, electrical, dielectric and magnetic properties of dysprosium doped Cobalt-Nickel ferrites and their response to gamma irradiation. Nucl. Instrum. Methods Phys. Res., B.

[cit61] Banaj L., Agrawal S. (2023). Dielectric behavior of Zr4+ doped MgFe2O4 spinel ferrite synthesized by solid-state reaction method. Curr. Appl. Phys..

[cit62] Hakeem A. (2021). *et al.*, Magnetic, dielectric and structural properties of spinel ferrites synthesized by sol-gel method. J. Mater. Res. Technol..

[cit63] An J.-S. (2023). *et al.*, Unveiling of interstice-occupying dopant segregation at grain boundaries in perovskite oxide dielectrics for a new class of ceramic capacitors. Energy Environ. Sci..

[cit64] Pawar R. (2017). *et al.*, Inter-atomic bonding and dielectric polarization in Gd3+ incorporated Co-Zn ferrite nanoparticles. Phys. B.

[cit65] Almond D., Duncan G., West A. (1983). The determination of hopping rates and carrier concentrations in ionic conductors by a new analysis of ac conductivity. Solid State Ionics.

[cit66] Fawzi A. S., Sheikh A., Mathe V. (2010). Structural, dielectric properties and AC conductivity of Ni (1−x) ZnxFe2O4 spinel ferrites. J. Alloys Compd..

[cit67] Oumezzine E. (2017). *et al.*, Frequency and temperature dependence of conductance, impedance and electrical modulus studies of Ni0. 6Cu0. 4Fe2O4 spinel ferrite. J. Alloys Compd..

[cit68] Mohapatra P. P., Pittala S., Dobbidi P. (2020). Temperature dependent broadband dielectric, magnetic and electrical studies on Li1-xMg2xFe5-xO8 for microwave devices. J. Mater. Res. Technol..

[cit69] Rout A., Agrawal S. (2022). Structural, morphological and electrical properties of new type Dy doped Ca6-xNa2Y2 (SiO4) 6 (OH) 2 hydroxyapatite compound synthesized by Co–precipitation method. J. Electroceram..

[cit70] Kamran M., Anis-ur-Rehman M. (2022). Resistive switching effect in RE-Doped cobalt ferrite nanoparticles. Ceram. Int..

[cit71] Kamran M., Anis-ur-Rehman M. (2020). Enhanced transport properties in Ce doped cobalt ferrites nanoparticles for resistive RAM applications. J. Alloys Compd..

[cit72] Jebli M. (2021). *et al.*, Structural and morphological studies, and temperature/frequency dependence of electrical conductivity of Ba 0.97 La 0.02 Ti 1− x Nb 4x/5 O3 perovskite ceramics. RSC Adv..

[cit73] Dhanya S., Satapathy J., Kumar N. P. (2023). Electrical properties of (Nd, Cr) co-doped Bismuth Ferrites synthesized via solid state method. Mater. Sci. Eng. B.

[cit74] Ali A. I. (2023). *et al.*, Dielectric and dynamic antibacterial investigations of organic–inorganic conductive membranes based on oxidized cellulose with BNKT nanoceramics. Cellulose.

[cit75] Khosrozadeh M. (2024). *et al.*, Complex impedance spectroscopy, dielectric response, and magnetic properties of the La0. 7 Sr0. 3BO3 (B= Mn, Fe, Co, or Ni) perovskite oxides. Ceram. Int..

[cit76] Mahapatro J., Agrawal S. (2021). Effect of Eu3+ ions on electrical and dielectric properties of barium hexaferrites prepared by solution combustion method. Ceram. Int..

[cit77] Butt K. Y. (2021). *et al.*, The study of structural, magnetic and dielectric properties of spinel ferrites for microwave absorption applications. Appl. Phys. A.

[cit78] Almessiere M. A. (2021). *et al.*, Electrical and dielectric properties of rare earth substituted hard-soft ferrite (Co0. 5Ni0. 5Ga0. 01Gd0. 01Fe1. 98O4) x/(ZnFe2O4) y nanocomposites. J. Mater. Res. Technol..

[cit79] Acharya N., Sagar R. (2021). Structure and electrical properties characterization of NiMn2O4 NTC ceramics. Inorg. Chem. Commun..

[cit80] Stein C. (2018). *et al.*, Structural and magnetic properties of cobalt ferrite nanoparticles synthesized by co-precipitation at increasing temperatures. AIP Adv..

[cit81] Allaedini G., Tasirin S. M., Aminayi P. (2015). Magnetic properties of cobalt ferrite synthesized by hydrothermal method. Int. Nano Lett..

[cit82] Yuan G. (2006). *et al.*, Structural transformation and ferroelectromagnetic behavior in single-phase Bi1− xNdxFeO3 multiferroic ceramics. Appl. Phys. Lett..

[cit83] Wang D. (2015). *et al.*, Sol–gel synthesis of Nd-doped BiFeO3 multiferroic and its characterization. Ceram. Int..

[cit84] Venkat U., Seenuvasakumaran P. (2023). Synthesis, structural, micro structural, optical, magnetic and dielectric properties of Nd doped multiferroic bismuth iron oxide. Results Mater..

[cit85] Abbas S. S. (2015). *et al.*, Ce-substituted Co 0.5 Ni 0.5 Fe2O4: Structural, morphological, electrical, and dielectric properties. Electron. Mater. Lett..

